# When assessing sleep performance in dialysis patients, objective measures of sleep quality should be preferred to the PSQL

**DOI:** 10.12669/pjms.41.12.13971

**Published:** 2025-12

**Authors:** Josef Finsterer

We read with interest the article by Parmaksiz et al. on an observational cross-sectional study of sleep quality, measured using the Pittsburgh Sleep Quality Index (PSQI), in 51 patients who had been receiving hemodialysis (HD) for more than one year and 23 patients who had been receiving peritoneal dialysis (PD) for more than one year.^1^ There was no difference in sleep quality between HD and PD patients in the PSQI, but HD patients had a longer sleep onset latency and more severe daytime disturbances compared to PD patients.^1^ It was concluded that sleep disturbances in dialysis patients should be treated.^1^ The study is interesting, but some points should be discussed.

The first point is that sleep quality depends not only on whether the patient receives HD or PD treatment, but also on several other endogenous or exogenous factors.^2^ Endogenous factors that additionally influence sleep performance include personality type, acute and chronic stress (balance between the sympathetic and parasympathetic nervous systems), the ability to cope with exogenous or endogenous stressors, genetic predisposition, comorbidities, and lifestyle habits (e.g., sleep habits (going to bed at a specific time, turning off the TV, headphones, radio, cell phone, iPad, and bedroom lights, removing stale air and all devices that generate electrosmog, and turning them off). Exogenous factors that influence sleep quality include noise, light, vibrations, drafts, temperature, humidity, insects, pets, earthquakes, bed, children, partner, electrosmog, cell phone towers, relationships with neighbors, socioeconomic status, time of last meal or fluid intake, concomitant medications, consumption of alcohol, adrenergic stimulants, or illegal dietary supplements, as well as local and geopolitical stress.^2^ As long as these determinants of sleep are not taken into account and included in the analysis, the data presented may be misleading.

The second point is that the PSQI has limitations regarding the assessment of sleep quality.^3^ The PSQI relies on subjective assessment and has a weak correlation with objective sleep measures. Other limitations include inconsistent results across different populations, limited meaningfulness in those who have difficulties understanding or writing, poor test-retest reliability, and missing guidelines regarding the test-retest intervals.^3^

The third point is that the group sizes were small in both the HD and PD groups. The small group sizes limit the significance of the study, which is why the study should be repeated with larger groups and with the inclusion of patients from different centers. It should also be ensured that the group sizes in the HD and PD groups are similar.

The fourth point is that concomitant medications and comorbidities were not sufficiently included in the analysis.^1^ Sleep quality can be impaired not only by dialysis, but also by psychiatric disorders (depression, anxiety, post-traumatic stress disorder, psychosis, mania, autism spectrum disorder), central nervous system disorders (epilepsy, dementia, Parkinson’s disease), developmental disorders, sleep apnea syndrome, cardiovascular diseases, restless legs syndrome, chronic pain syndromes, and polyneuropathy.^4^ In addition, various medications can disrupt sleep,^5^ which is why it is essential to include current medication in the interpretation of the results.

The fifth point relates to the discrepancy between the “Methodology” section, which states that all patients taking hypnotics were excluded, and the “Results” section, where Table-I shows that at least some of the patients included in both groups were taking hypnotics.^1^ This discrepancy should be resolved.

Overall, sleep disorders in dialysis patients should be assessed objectively using polysomnography, as the PSQI has several limitations that can lead to misinterpretation.

**KEYWORDS:** Sleep quality, PSQI, Dialysis, Sleep latency, Sleep architecture.
